# The neuro-cardio-facial-cutaneous syndrome – unity in diversity

**DOI:** 10.1186/1687-9856-2013-S1-P205

**Published:** 2013-10-03

**Authors:** Carmen Vulpoi, Mihaela Anton, Ion Poeata, Jeanina Idriceanu, Cristina Rusu

**Affiliations:** 1 University Of Medicine And Pharmacy, Grigore.T.Popa, Romania; 2 Regional Hospital Bacu, Romania

## 

The neuro-cardio-facial-cutaneous syndrome (NCFCS) concept was recently established in order to group a number of hereditary disorders characterized by a variable degree of growth and mental retardation, cardiac defects, dysmorphic facial features and skin abnormalities, and having a common background, germline mutations in genes of the RAS-MAPKinase pathway. The included entities are Noonan and Leopard syndrome (the most frequents), Costello syndrome, cranio-facio-cutaneous syndrome, as well as some forms of type 1 neurofybromatosis and the newly defined Legius syndrome.

We present illustrative cases of 2 of this syndromes, Noonan (NS) and Leopard syndrome (LS), illustrative for both the common elements and the variety of the characteristics. Four of the six cases of Noonan syndrome are treated with growth hormone, with a good response, proving the importance of an early diagnostic. Two cases are fronm the same family (mother and son), the mother of another case has patognomic features but the diagnostic had not yet been confirmed. One of the children with NS, recently diagnosed, with important vertebral deformation needing specific treatment cannot, for the moment, be treated with hGH in spite of the important growth delay (>-3SD).

LEOPARD is an acronym for the major features of the disorder: Lentigines, ECG conduction abnormalities, Ocular hypertelorism, Pulmonary stenosis, Abnormal genitalia, Retardation of growth, Deafness. The probant case of LS, a 15 years old boy, presented almost all these features, with the exception of deafness. He associated a cerebral tumor, rare in LS, but which could be considered as a NCFCS feature, the MAPK pathway being involved in tumorigenesis. He presented with short stature and neurologic symptoms, both improved after the (partial) tumor resection (pylocytic astrocytoma). The genetic investigation of the family confirmed the syndrome at 4 other members (father, two brothers and a sister), each one with various manifestations, from only café-au-lait spots to cardiac malformations.

The absence of patognomonic symptoms as well as the overlap of numerous features makes the diagnosis of the components of NCFCS difficult, the molecular diagnosis offering the chance to exceed the clinical difficulties. More than that, specific mutations are associated with specific fenotypes, which has a great importance for the diagnostic and prognostic. An early diagnostic is important not only for the rapid treatment of life threatening elements (cardiac malformations, tumors) and chronic treatment of some features (like short stature), but also for an appropriate genetic counseling.

